# Not Lupus Nephritis but a Rare Case of Drug-Induced Pauci-Immune Glomerulonephritis

**DOI:** 10.7759/cureus.21549

**Published:** 2022-01-24

**Authors:** Stephanie Hang, Priyadarshini Dixit, Sarah Fatima, Dilnaz Alam, Christopher Webster

**Affiliations:** 1 Internal Medicine, St. Joseph Mercy Oakland Hospital, Pontiac, USA; 2 Nephrology, St. Joseph Mercy Oakland Hospital, Pontiac, USA

**Keywords:** acute renal fail, hydralazine-induced lupus syndrome, hydralazine, pauci-immune crescentic glomerulonephritis, drug-induced-lupus

## Abstract

Hydralazine-induced pauci-immune glomerulonephritis is a rare cause of glomerulonephritis. It is an anti-neutrophil cytoplasmic antibody (ANCA) associated vasculitis that can be rapidly progressive and potentially life-threatening. However, most cases are found to be asymptomatic, and patients often present with acute renal failure and painless hematuria. It has been confused with lupus nephritis but treatment differs, thus, necessitating the need for differentiation.

A case report of an 80-year-old African American woman with a history of hypertension, diabetes mellitus type 2, and hypothyroidism, who presented with generalized weakness and weight loss of 30-40 lbs. The patient had been treated with hydralazine for months for hypertension. She presented to the hospital with acute renal failure that worsened over the course of several months eventually requiring hemodialysis.

The patient was found to have drug-induced ANCA vasculitis from hydralazine. This etiology was confirmed with pauci-immune glomerulonephritis seen on renal biopsy. This presentation has the potential of being confused with lupus nephritis. Despite the initial serology being suggestive of lupus, this type of nephritis does not have positive immunofluorescence.

The treatment of nephritis in this patient was generally supportive. However, it was important to identify the underlying cause of renal failure. Equally important to initiating immunosuppressive therapy, it was imperative to discontinue the offending drug in a timely manner to prevent rapid organ failure. The causative agent, hydralazine, may have otherwise gone unnoticed without a thorough investigation into other causes of renal failure. Thus, it is important to consider this as a diagnosis with a patient who presents with rapidly progressive renal failure on hydralazine and may mimic lupus nephritis.

## Introduction

This case report was presented at National ACP Meeting in April 2021 and ACP Michigan Chapter Residents Day in May 2021 with the same authors.

Glomerulonephritis is the inflammation of the glomeruli in the kidneys and can be further classified based on the pathogenesis - immune complex deposits, anti-glomerular basement membrane antibodies, and anti-neutrophil cytoplasmic antibodies (ANCAs) or small vessel vasculitis [1-4]. The course and presentation of these disease processes vary, but often present as worsening serum creatinine, hematuria, and/or proteinuria [5]. More specifically, a subclass called rapidly progressive glomerulonephritis (RPGN) is characterized by extensive crescent formation in the glomeruli as a response to injury to the capillary wall and leads to loss of renal function. RPGN can occur secondary to immune complex medicated injury, anti-glomerular basement membrane antibody disease, or pauci-immune necrotizing and crescentic glomerulonephritis.

Pauci-immune glomerulonephritis (PIGN) is a rare cause of glomerulonephritis which can be life-threatening. It is associated with minimal or absence of immune deposits, which may be limited to the renal vessels, and can be divided into ANCA positive or ANCA negative. Drug-induced vasculitis is an example of ANCA positive PIGN and can occur with hydralazine, disease-modifying anti-rheumatological drugs, allopurinol, propylthiouracil, and phenytoin [6,7]. The majority of cases have high titers of myeloperoxidase ANCA antibodies, while others may also have other immunological antibodies which become challenging for providers to establish the diagnosis. PIGN shows no or minimal evidence of immunofluorescence on renal biopsy, as in ANCA-associated glomerulopathies. Most cases are found to be asymptomatic and have been confused with lupus nephritis.

This case highlights the importance of prompt recognition of the disease and the need for immediate withdrawal of the culprit to preserve renal function. The underlying diagnosis was masked due to the patient’s clinical presentation and associated co-morbidities.

## Case presentation

An 80-year-old African American woman with a history significant for hypertension, diabetes mellitus type 2, and hypothyroidism presented with generalized weakness and weight loss of 30-40 lbs over a period of 3 months. She was on hydralazine for hypertension for 6 months. She did not use tobacco, alcohol, or illicit drugs. Physical examination was remarkable for alopecia and non-pitting edema. Laboratory evaluation on admission revealed an elevated blood urea nitrogen (BUN 36 mg/dL), creatinine (Cr 2.21 mg/dL) demonstrating acute kidney injury (AKI) with a baseline creatinine of 1.5 to 1.7 prior to admission. The patient had bicytopenia (WBC 2,200/mcL and hemoglobin 7.4 g/dL) as well as hematuria with proteinuria found on urinalysis with reduced urine output.

**Figure 1:**
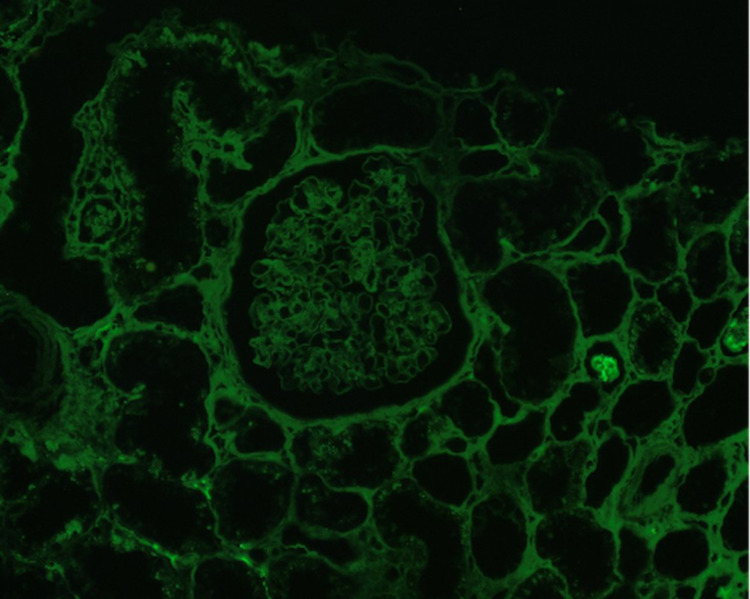
Immunofluorescence is negative for IgM, C3, Kappa, and Lambda confirming PIGN PIGN - Pauci-immune glomerulonephritis

**Figure 2:**
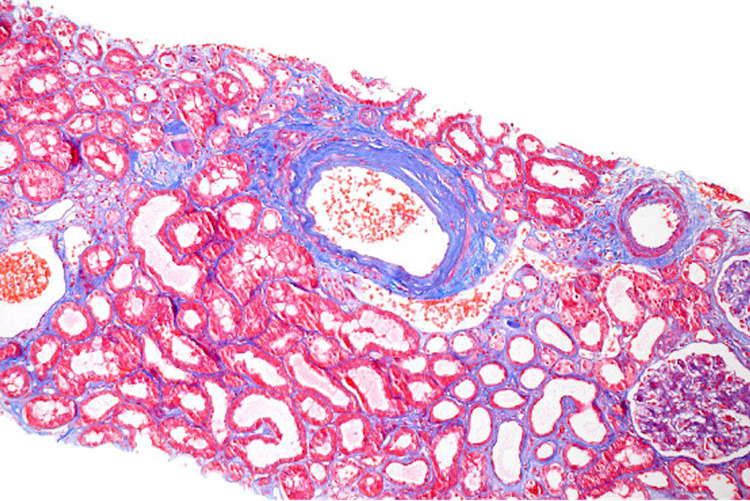
Acute tubular injury with arteriosclerosis

During the hospital course, she developed worsening renal function leading to acute renal failure with BUN 86 mg/dL and Cr 6.73 mg/dL. The primary team initially thought it was lupus nephritis, which prompt further serologic workup. Autoimmune workup was positive for ANA, proteinase 3 ANCA, myeloperoxidase ANCA, low complements, and anti-dsDNA. Also, hepatitis B surface antibody was reactive consistent with immunity. Based on the above results it was still thought to be lupus nephritis. The patient was diagnosed with drug-induced ANCA vasculitis only after a biopsy. This etiology was confirmed with pauci-immune glomerulonephritis seen on biopsy (Figures 1, 2). The serology work-up was a red herring to the team in order to have lupus nephritis the biopsy must show positive immunofluorescence. The patient was treated with RAVE protocol of rituximab and pulse steroids with discontinuing hydralazine. Ultimately, she was discharged on hemodialysis, maintenance steroids, and asked to follow up with nephrology as an outpatient.

## Discussion

Hydralazine is a commonly used vasodilator, often used for resistant hypertension. Hydralazine-induced vasculitis or Drug-induced PIGN is associated with dual ANCA positivity and sometimes associated with anti-nuclear (ANA) or anti-dsDNA antibodies. The incidence of hydralazine-induced ANCA-mediated vasculitis is proportional to the dose and duration of use. The incidence rate can be up to 10.4% in patients taking a dose of 200 mg/day for greater than three years [8]. Serum complement levels can be beneficial in differentiating the underlying pathogenesis since C3 and C4 levels are usually normal in PIGN and anti-GBM disease as opposed to the low levels seen in another glomerulonephritis [9,10]. The serological studies may also cause confusion like in our case. Ultimately, a biopsy will confirm the diagnosis (seen in Figures 1, 2), which will have immunofluorescence negative for IgM, C3, Kappa, and lambda.

In contrast, lupus nephritis can occur in patients with systemic lupus erythematosus, due to the deposition of immune complexes mainly comprising anti-double-stranded DNA antibodies [7]. In many patients’ lupus nephritis may be an initial presentation of SLE. Lupus nephritis can have a similar presentation to drug-induced vasculitis with similar overlapping characteristics of elevated serum creatinine in the presence of elevated anti-dsDNA titers and low complement levels. Also in this case, due to ANA positivity with the nuclear pattern: mixed homogenous and speckled, which is mostly seen in SLE. Therefore, these laboratory findings appear indicative of lupus nephritis but the utility of serological assessment differs among patients. These findings may result in misdiagnosis. It is important to make a diagnosis on the biopsy and the immunofluorescence results.

It is important to not have a linear approach to treatment. Drug-induced PIGNs are rare, but it’s necessary to have a high level of suspicion, especially in patients with multiple comorbidities and varying symptoms. There is a higher incidence of drug-induced nephropathy in patients who are greater than 60 years old, patients with diabetes, heart failure, and those with pre-existing renal insufficiency [7,8]. It has been reported it is more common in females and patients with thyroid disease with 80% of these patients having renal involvement on initial presentation [5]. It can be easily missed or even confused with other diseases such as lupus [5]. There are few or no immune deposits visualized in PIGNs whereas immunofluorescence with multiple immune deposits in lupus nephritis. This is one of the most crucial distinctions between drug-induced vasculitis and lupus nephritis. It has a similar serologic profile to lupus, in which ANCA-associated glomerulonephritis can have positive ANA or other positive lupus serology. This can result in inappropriate treatment with the continuation of the offending drug.

## Conclusions

We present a case with many distractors as the patient’s symptoms were unrelated to the patient’s final diagnosis. Almost equally important as the immunosuppressive treatment, is to identify and discontinue the offending drug in a timely manner to prevent rapid organ failure. The causative agent, hydralazine, may have otherwise gone unnoticed without the prompt and dedicated work of the medical team as the patient had multiple distractors present.
